# Exploring the Influence of *Fok1*/*Apa1* Polymorphic Variants on Adolescent Mental Health and Response to Vitamin D Supplementation in Embryonic Hippocampal Cell Lines

**DOI:** 10.3390/genes15070913

**Published:** 2024-07-12

**Authors:** Giulia Gizzi, Federico Fiorani, Samuela Cataldi, Martina Mandarano, Elisa Delvecchio, Claudia Mazzeschi, Elisabetta Albi

**Affiliations:** 1 Department of Philosophy, Social Sciences and Education, University of Perugia, 06126 Perugia, Italy; elisa.delvecchio@unipg.it (E.D.); claudia.mazzeschi@unipg.it (C.M.); 2 Department of Pharmaceutical Sciences, University of Perugia, 06126 Perugia, Italy; federico.fiorani@dottorandi.unipg.it (F.F.); samuela.cataldi@gmail.com (S.C.); 3 Division of Pathological Anatomy and Histology, Department of Medicine and Surgery, University of Perugia, 06126 Perugia, Italy; martina.mandarano@unipg.it

**Keywords:** *Fok1*, *Apa1*, anxiety, depression, vitamin D, vitamin D receptor

## Abstract

Several single nucleotide polymorphisms (SNPs) of the vitamin D receptor (VDR) have been observed in association with susceptibility to various pathologies, including autism, major depression, age-related changes in cognitive functioning, and Parkinson’s and Alzheimer’s diseases. This study aimed to establish the association between *Fok1*/*Apa1* polymorphic variants and anxious/depressive symptoms in nonclinical adolescents from central Italy, with the goal of identifying the risk of developing both symptoms. We found no significant difference in genotype distribution or dominant/recessive models of *Fok1*/*Apa1 VDR* polymorphic variants between subjects with anxious/depressive symptoms and controls. HN9.10e cell lines carrying the AA genotype for *Fok1* and the CC genotype for *Apa1* responded better to treatment with vitamin D3 than cell lines carrying the AG genotype for *Fok1* and CA genotype for *Apa1*. Cell lines carrying the GG genotype for *Fok1* and the AA genotype for *Apa1* did not respond at all, suggesting avenues for future studies in both the general population and individuals with mental and/or neuropsychiatric disorders. These studies suggest that the level of response to vitamin D3 administered to prevent and/or treat mental or neurological disorders could depend on the polymorphic variants of the vitamin D receptor.

## 1. Introduction

It is known that active vitamin D (VitD) or calcitriol is derived from liver and renal hydroxylation of cholecalciferol, formed in the skin under the influence of the UVB portion of the light spectrum of sunlight. Additionally, VitD can also be derived from animal (VitD3) or vegetable (VitD2) dietary products [1] or can be taken as a supplement [2]. Exogenous VitD is transported in the blood, bound to vitamin D binding protein (DBP) and enters cells expressing the megalin/cubilin complex, which facilitates its transport inside the cells [3]. Both endogenous and exogenous VitD can have non-genomic and genomic effects [4]. As a consequence, exogenous VitD is considered a nutrient with a nutrigenomic effect [5]. The non-genomic effect of VitD is due to its interaction with one of its receptors located in the cell membrane or cytosol [6], activating signal mediators such as cyclic AMP and diacylglycerol, and inositol phosphate derived from phosphoinositide hydrolysis [7]. Otherwise, the genomic effect is dependent on the VitD interaction with the nuclear receptor (VDR), which is also a transcription factor [3]. When the ligand binds to the VDR, histone modifications are induced, resulting in a change in chromatin accessibility [8]. Therefore, the genomic system is based on the existence of VDR, on its ligand (active VitD), and on numerous proteins and enzymes that are activated downstream of the VitD/VDR axis. As a consequence, there is modulation of the transcription of hundreds of target genes [5]. The VDR is known to reside in specific lipid microdomains located in the inner nuclear membrane [9]. Accurate assessment of VDR function is critical to understanding the response to active VitD in various pathophysiological conditions. The VitD/VDR axis, originally known for controlling calcium/phosphorus metabolism in the bone, is now considered essential for the functionality of numerous organs and tissues such as muscle, skin, adipose tissue, the immune, cardiorespiratory, gastrointestinal, and, last but not least, most importantly, the central nervous system [10,11,12]. It is known that the expression of VDR in the brain changes in different experimental conditions. In an experimental model of contextual fear conditioning in rats used to understand fear and anxiety in humans, VDR was upregulated in the prefrontal region [13]. Differently, VDR was downregulated in a mouse experimental model of Niemann–Pick Type A Disease [14] and Parkinson’s disease [15].

The VDR belongs to the nuclear receptor superfamily in which members have close structural similarity, suggesting the possibility of a single evolutionary origin [16]. Variations in the *VDR* gene located in chromosome 12 (12q13.11) induce changes in receptor expression and function. To date, several single nucleotide polymorphic variants (SNP) of the *VDR* have been observed in association with the susceptibility of different pathologies [17,18,19]. SNP *Fok1* (*rs2228570*), *Apa1* (*rs7975232*), *Bsm1* (*rs1544410*)*,* and *Taq1* (*rs731236*) are the most studied. Interestingly, *Fok1* (*rs2228570* G>A), located in exon 2, and *Apa1* (*rs7975232* C>A) in intron 8 [20] were associated with neuropsychiatric disorders [21,22,23]. Of note, *Fok1* was associated with epilepsy [20], Parkinson’s disease [22,23], hyperactivity behavior in children with autism spectrum disorder [24,25], and schizophrenia [26]. Moreover, *Apa1* was associated with Alzheimer’s disease [27], and both *Fok1* and *Apa1* influenced the susceptibility to age-related changes in cognitive functioning and depressive symptoms [28].

There is currently no study on the relationship between *VDR* gene polymorphic variants and anxiety/depression symptoms in nonclinical patients and the response of cell lines to vitamin D treatment. The aim of the study was to evaluate the presence of anxious and/or depressive symptoms in non-clinical adolescents during the COVID-19 period and to establish a possible relationship with the *Fok1/Apa1* polymorphic variants of the *VDR*. Furthermore, the next objective was to evaluate whether the response to treatment with VitD3 differs in the analyzed cell lines with different *Fok1* and *Apa1* polymorphic variants of the *VDR* gene polymorphic variant.

## 2. Results

### 2.1. VDR Polymorphic Variant in Adolescents

To explore the distribution of *VDR* polymorphic variants in 184 non-clinical Italian adolescents, *Fok1* (*rs2228570*) and *Apa1* (*rs7975232*) were analyzed in the total sample in males and females.

As shown in Table 1, the prevalence of the AA genotype (42.9%) was higher than the GG genotype (9.8%) for *Fok1,* and the CC genotype (31.0%) was higher than the AA genotype (25.0%) for *Apa1* in the total population. About half of the population showed heterozygosity (47.3% AG for *Fok1* and 44.0% CA for *Apa1*). Thus, in the population under study, A was the dominant allele for *Fok1*, and C was the dominant allele for *Apa1*.

Among all the participants, 68 were male (M) and 116 female (F) (Table 1). By analyzing the distribution of sex in relation to the genotypes, it was evident that AA was 38.0%M and 62.0%F, GG was 27.8%M and 72.2%F, and AG 37.9%M and 62.1%F for *Fok1* (Table 1). Statistical analysis indicated that the differences were not significant (*χ^2^*_(5)_ = 0.722, *p* = 0.70). Similarly, for *Apa1*, CC was 42.1%M and 57.9%F, AA was 37.0%M and 63.0%F, and CA was 33.3%M and 66.7%F (*χ^2^*_(5)_ = 1.105, *p* = 0.58).

In order to ascertain whether there was a variation in polymorphic variants based on parental origin, 184 participants were divided into those with Italian parents (n = 150) and those with foreign parents (n = 34). No statistically significant differences were found between the two groups when considering the genotype distribution of *Fok1* (*χ^2^*_(2)_ = 2.935, *p* = 0.23) and *Apa1* (*χ^2^*_(2)_ = 1.159, *p* = 0.56). The possibility that there could be differences if the number of adolescents with Italian parents and foreign parents were identical cannot be ruled out. However, it is difficult to achieve this goal in the Umbrian schools that participated in the study.

Furthermore, no significant variation was evident when considering only fathers or mothers of foreign origins. However, for *Fok1*, individuals with foreign parents—especially foreign fathers—exhibited a higher prevalence of the AA genotype compared to those with Italian parents (Table 2). Furthermore, with reference to *Apa1*, where the CC genotype prevailed in adolescents with Italian parents, there was an increase in the prevalence of the AA genotype in adolescents with foreign parents, particularly those with a foreign father (Table 2).

### 2.2. Difference in Depressive and Anxious Symptoms in Relation to Fok 1 and Apa1 Polymorphic Variants

Subsequently, the possible relationship between *Fok1* and *Apa1* polymorphic variants and the presence of anxious and/or depressive symptoms in the adolescents participating in the study was investigated. Firstly, the subjects with depressive and anxious symptoms versus the subjects without symptoms in males and females of the total sample, or in relation to age, were analyzed. The depressive symptoms and anxious symptoms were studied separately. Table 3 shows that a higher number of the females presented depressive and anxious symptoms than the males. Moreover, they appeared mainly at 16 and 17 years of age in the males, with the exception of anxious symptoms at 14 years of age, while in females, both symptoms appeared as early as 14 years of age, and the percentage of affected subjects remained similar over time.

The data shows that the percentages of subjects with depressive symptoms and those with anxious symptoms were similar whether males or females were analyzed.

Thus, the presence of depressive symptoms was assessed in subjects who were found to have anxious symptoms and the presence of anxious symptoms in subjects who were found to have depressive symptoms. Table 4 shows that about 62% of the subjects had both anxious and depressive symptoms, indicating a simultaneous presence of the two symptoms in the same subjects.

Thus, the association of depressive and/or anxious symptoms with *Fok 1* polymorphic variants was studied on genic models. These include an “allelic model”, “dominant model”, and “recessive model”. The “allelic model” allows an analysis of the comparison between the dominant allele in homozygosis (AA), the recessive allele in homozygosis (GG), and the alleles in heterozygosis (AG). The “dominant model” analyzes the comparison between the dominant homozygous allele + the heterozygous allele vs. the recessive homozygous allele. The “recessive model” analyzes the recessive allele in homozygous + the allele in heterozygous vs. the dominant allele in homozygous.

In the analysis of the allelic models (AA, GG, and AG genotypes), no value was statistically significant (Table 5a). Thus, to better investigate the relationships, the dominant model (AA + AG vs. GG) and the recessive model (GG + AG vs. AA) were investigated. In no case were statistically significant values found (Table 5b). Similar results were obtained by analyzing the *Apa1* polymorphism. No statistically significant differences were found with the allelic model (CC, AA, AC), with the dominant model (CC + CA vs. AA), or with the recessive one (AA + CA vs. CC) (Table 5a).

### 2.3. VDR Polymorphic Variant in Embryonic Hippocampal Cell Lines

To ascertain whether the *VD*R polymorphic variants might be related to the response to vitamin D treatment in brain cells, *Fok1* and *Apa1* polymorphic variants were assessed in embryonic hippocampal cell lines (HN9.10e). This cell line was chosen due to our previous experience that demonstrated how VitD3 induced differentiation towards both neurons and astrocytes. Therefore, it was important to evaluate the *VDR* polymorphism. As a control, two other completely different cell lines (keratinocytes, HaCat; and breast cancer cell lines, MCF7) were considered. The results revealed that the HN9.10e cell lines exhibited the *Fok1/Apa1* genotype AA–CC, the HaCat cell lines exhibited AG–AC, and the MCF7 cell lines exhibited GG–AA (Table 6).

To compare the cellular polymorphic variants with those of the adolescents under study, the percentage of subjects who simultaneously carried the AA genotype for *Fok1* and CC for *Apa1*, of those who carried AG for *Fok1* and CA for *Apa1*, and finally of those who carried GG for *Fok1* and AA for *Apa1* were considered. In the general population, AA + CC represented 12.4%, AG + CA 18.9%, and GG + AA 2.7%. In the subjects who presented depressive symptoms, AA + CC represented 6.4%, AG + CA 9.2%, and GG + AA 1.6% of the general population. The subjects who presented anxiety symptoms were 5.9% AA + CC, 9.7% AG + CA, and 1.0% GG + AA in the general population.

### 2.4. Effect of Vitamin D3 on Cell Lines with Different VDR Polymorphic Variants

VitD3 has been implicated in many cellular functions with both genomic and non-genomic activity [1]. Among the main genomic effects, VitD3 can regulate the gene expression of its own receptor, the VDR [11]. Thus, we treated the three cell lines reported above that have different genotypes of *VDR* polymorphic variants with VitD3 to determine whether the cell lines respond similarly, regardless of the polymorphic variants.

**Figure 1:**
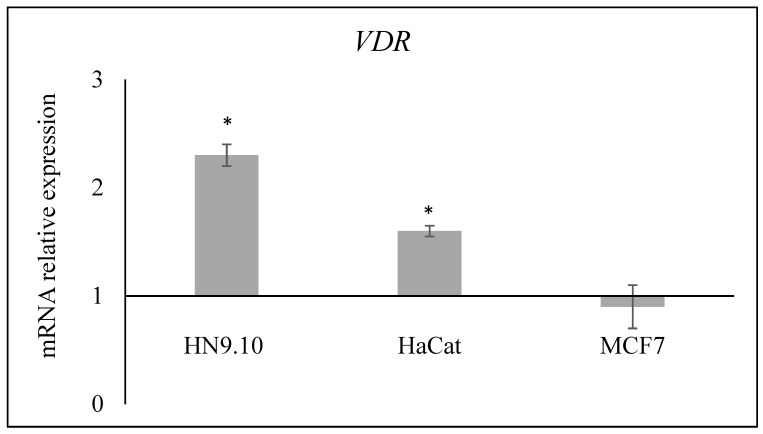
Effect of 100 nM VitD3 treatment on *VDR* gene expression in HN9.10e, HaCat, and MCF7 cell lines. GAPDH and 18S rRNA were used as housekeeping genes. Relative mRNA expression levels were calculated as 2^−ΔΔCt^, comparing the results of the treated samples with the untreated sample (control sample) equal to 1, the origin of the axes. Data are expressed as the mean ± SD of 3 independent experiments performed in duplicate. * *p* < 0.05 versus control sample.

An amount of 100 nM of VitD3 was added to a culture medium for 24 h. The results showed that only the HN9.10e and HaCat cell lines displayed an increase in the expression of the *VDR* gene, suggesting that cell lines carrying the AA–CC or AG–CA *Fok1*/*Apa1* genotypes are particularly sensitive to VitD3 treatment (Figure 1).

**Figure 2:**
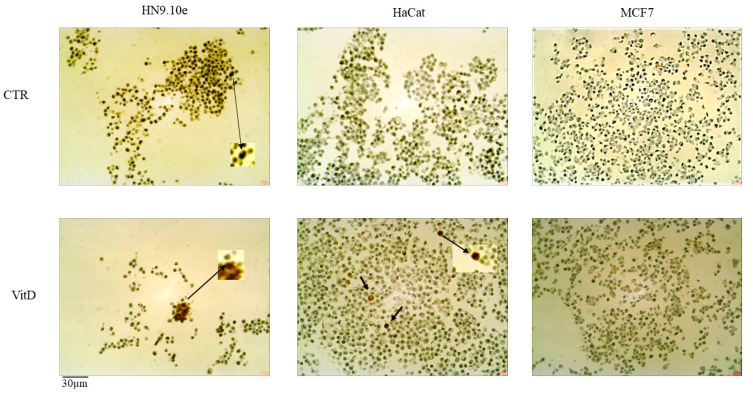
Response of the cell lines to 100 nM Vitamin D (VitD) treatment. The images show the result of the immunocytochemical analysis of Neurofilament 200 kDa (NF200) in embryonic hippocampal cells (HN9.10e cell lines), of Vimentin in keratinocytes (HaCat cell lines), and of HER2 in breast cancer cells (MCF7cell lines). The arrows indicate the positivity of the labeling studied, as reported in the 4.10 section. The lighter windows reveal greater detail in the enlarged images.

To establish the effect of the VitD3–VDR response in each cell line, the markers of differentiation were analyzed after VitD3 treatment for 24 h (Figure 2). For the HN9.10e cell line, the NF200, a marker for heavy neurofilament formation, was studied. The results showed very low staining in the control sample (8 ± 2%) and an increase in positivity to 25 ± 4% in the VitD3-treated cells. Vimentin was used to study the epithelial–mesenchymal transition in the HaCat cell line. No staining was evident in the control sample, and 12 + 3% positive cells appeared after the VitD3 treatment. The MCF7 cells were HER2-negative cell lines, and no changes in protein expression were evident after the VitD3 treatment.

## 3. Discussion

Our work addressed two different issues related to the *Fok1* and *Apa1* polymorphic variants of the VDR: (1) establishing whether the two polymorphic variants are related to the anxious and/or depressive symptoms of non-clinical adolescents; (2) evaluating whether cell lines with different *Fok1* and *Apa1* polymorphic variants respond in the same way to treatment with VitD3. The idea arose from the lack of studies on the relationship between *VDR* polymorphism and the effects of VitD3. The sample of non-clinical adolescents was chosen to avoid entering the field of pathology in which studies become more complex due to the coexistence of different triggering factors and pathogenetic mechanisms. Stress was considered a risk factor for anxiety and depression. Interestingly, the hippocampus as a center for cognition and memory is implicated in anxiety [29] and depression [30] modulation. The study started from data reported in the literature on the existence of a connection between VitD3 levels or *VDR* polymorphism and mental and/or neuropsychiatric disorders. Some studies suggested a link between *Fok1*/*Apa1* polymorphic variants and cognitive decline and depression in the elderly [28], Parkinson’s disease [23], Alzheimer’s disease [27], and schizophrenia [26]. Additionally, Lye et al. [31] found an association between the *Apa1* polymorphic variant and the severity of major depressive disorder (MDD). Of note, the AA genotype increased 6.4 times, and the genotype CA increased 11.3 times in severe MDD compared to mild and moderate MDD.

Certainly, the study of the polymorphic variants in relation to a complete clinical evaluation could also provide information on the duration of the disease, its severity, and possible comorbidities. Lye et al. [32] showed increased odds of MDD with certain *VDR* haplotypes. Conversely, Bozdogan et al., by analyzing *Taq1*, *Fok1*, *Bsm1*, and *Apa1* polymorphic variants, found no significant differences between autism spectrum disorder patients and healthy controls [33]. Moreover, by analyzing the *Fok1* polymorphic variant, Can et al. showed no differences between patients with MDD and controls [34].

The objective of our study was to evaluate the presence of anxious and depressive symptoms in adolescents who did not have a diagnosis of anxiety or depression. The aim was to have a general picture in schools to develop in the future an intervention to prevent the appearance of these symptoms. Therefore, the psychological analysis was aimed at revealing symptoms and not making a diagnosis. The results were reported in each class for groups of participants and not for each individual participant. Furthermore, the data obtained were presented at conferences with the participation of the school director, teachers, and students.

When the project was designed, it was not known that there would be COVID-19. Certainly, the lifestyle changes that COVID-19 demands have had a significant impact on the mental health of adolescents, too. Therefore, the results of the study must be considered as data on the specific physical–psychological condition of the COVID-19 period. We demonstrated that, in the study population, the dominant allele is A for *Fok1* and C for *Apa1* and that the prevalent genotype for both is heterozygous (AG for *Fok1* and CA for *Apa1*). We found no statistically significant differences in the distribution of *Fok1* and *Apa1* genotypes based on sex or parental origin (Italian or foreign). Furthermore, no significant differences were observed in genotype distribution between adolescents with and without anxious and/or depressive symptoms, even when considered dominant or recessive models. The discrepancy with Lye et al.’s data [29] might be due to the fact that the adolescents participating in the study have depressive symptoms but do not have a diagnosis of MDD.

Thus, the main finding of our study was that *Fok1* and *Apa1* polymorphic variants of the *VDR* gene did not appear to contribute to the development of anxious and depressive symptoms in non-clinical adolescents. Excluding the relationship between *VDR* polymorphic variants and these specific symptoms was a negative but relevant result. A limitation of the article was the number of participants. However, we wanted to work with a very homogeneous population, adolescents aged 14 to 17 attending secondary schools in Umbria, Italy. In addition, the trial was carried out during the COVID-19 period, with great difficulty in recruiting patients. Also, it was difficult to get parental permission. Further studies are certainly needed.

The second interesting aspect of the study is that we tried to establish a possible correlation between *VDR* polymorphic variants and the response to treatment with VitD3. A limitation of the study was that we did not have any data on the blood levels of VitD3 in the participants. This was due to the difficulty of obtaining blood samples from adolescents in schools. However, even if all participants had a physiological blood level of VitD3, it would not have been possible to determine whether everyone responded to VitD3 in the same way, regardless of the polymorphism. So, we considered doing an in vitro study since the *Fok1 VDR* polymorphic variant influences the response to VitD supplementation [35].

We chose a brain cell line and a precise embryonic hippocampal cell line (HN9.10e) since we previously demonstrated how VitD*3* induced their differentiation towards both neurons and astrocytes. Therefore, it was important to evaluate the *VDR* polymorphic variants. As a control, two other completely different cell lines (keratinocytes, HaCat; and breast cancer cell lines, MCF7) were considered. Gleba et al. had already indicated that not the level of VDR but its polymorphic variations, especially *Fok1*, might be a factor that plays a role in the sensitivity of different cell lines (human leukemia and lymphoma cell lines) to VitD3 [36]. Additionally, Maj et al. studied *VDR* polymorphic variants in different cell lines showing that the cell line most resistant to the action of vitamin D3 (NCI-H358) was a recessive *rs2228570* (*Fok1*) homozygote [37]. Thus, we decided to study the polymorphic variants of *Fok1* and *Apa1* in three completely different cell lines, with the aim of evaluating the effect of VitD3 on the cell lines.

Interestingly, the three cell lines had different genotypes for both *Fok1* and *Apa1*, and to be precise, the HN9.10e cell line carried the AA genotype for *Fok1* and the CC genotype for *Apa1*, the HaCat cell line carried the AG genotype for *Fok1* and the CA genotype for *Apa1*, and the MCF7 cell line carried the GG genotype for *Fok1* and the AA genotype for *Apa1*. Thus, we had cell lines with different genotypes of the two polymorphic variants and could study whether the VitD3 effect was similar or not. It was demonstrated that VitD3 is a molecule with a nutrigenomic effect and determines the gene regulation of its own receptor [11]. So, we first studied the expression of the VDR gene. Our results indicated that the increase in *VDR* expression following VitD3 treatment was very high in the HN9.10e cell line, moderately elevated in the HaCat cell line, and absent in the MCF7 cell line. Therefore, cell lines with different *VDR* polymorphic variants responded differently to VitD3 treatment. The gene expression of the mRNA for the synthesis of VDR was a direct consequence of the interaction of VitD3 with receptors synthesized by the *VDR* gene with different polymorphic variants. So, we conducted a functional study to see if there were any biological consequences. In HN9.10e, by interacting with its overexpressed receptor, VitD3 induced an upregulation of the NF200 protein essential for neurite formation, according to previous results [12]. In some HaCat cells, VitD3 stimulated the vimentin expression by indicating their epithelial-mesenchymal transition [36]. No effect was evident for HER2 in breast cancer cells. Taken together, the results indicate that only cell lines presenting specific *VDR* polymorphic variants (AA *Fok1* and CC *Apa1* or AG *Fok1* and CA *Apa1*) were able to respond to VitD3 with molecular changes involved in specific cell functions. The possibility that the different responses of the three cell types to VitD3 treatment might also be independent of the VDR polymorphic variants cannot be excluded. Future studies on multiple cell lines and on VDR structure/function will be necessary.

The final effect on the markers can certainly also be multifactorial, but we demonstrated that cells respond differently depending on whether VitD3 was administered to cell lines with different gene polymorphic variants of its receptor. On the other hand, all cellular responses to treatment are the result of different metabolic mechanisms that are activated, and it is very difficult to establish a direct cause-effect relationship. Our data demonstrate that MCF7 cell lines with the GG–AA genotype, which did not increase *VDR* gene expression upon Vitamin D treatment, also did not have a functional cellular response. This is the first observational work of basic research. Subsequent studies might clarify the involved mechanisms and open the doors to numerous future works on the topic, from molecular biochemistry to therapeutics.

These findings suggest that individuals with AA (*Fok1*) and CC (*Apa1*) genotypes might respond better to VitD3 treatment, while those with the GG genotype for *Fok1* and AA for *Apa1* might not respond at all. In the participants of the study, the subjects carrying the GG genotype for *Fok1* and AA for *Apa1* represented a minority in both the general population (2.7%) and among the individuals with anxious (1%) and/or depressive (1.6%) symptoms. As a consequence, 96.2% of GG + AA carriers presented either depressive or anxious symptoms. Therefore, in the future, it will be necessary to expand the study to larger samples of different populations that perhaps have a higher percentage of the GG + AA polymorphism. The possibility that these subjects do not respond to treatment with VitD3 might be relevant for other disorders that go beyond anxious and/or depressive symptoms.

Thus, our study suggests that the *VDR* polymorphism should always be considered when analyzing associations between VitD3 levels and pathologies. In fact, the level of response to VitD3 administered to prevent and/or treat mental or neurological disorders could depend on the receptor polymorphic variants. It is difficult to demonstrate that internal levels in the blood depend on the receptor polymorphic variants because they are regulated by calcium and parathyroid hormones. It would be interesting to establish the correlation between the polymorphic variants of VDR and the effects of VitD3 in patients. The demonstration with cell lines with different polymorphic variants is only a first step. Further studies will be necessary to fully understand the association between *VDR* gene polymorphic variants and the response to VitD3.

## 4. Materials and Methods

### 4.1. Experimental Plan

**Figure 3:**
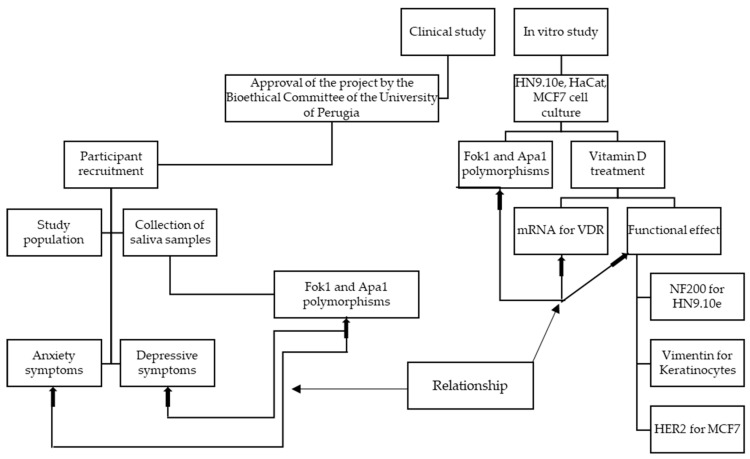
Experimental design with clinical and in vitro studies.

The experimental plan is illustrated in the following diagram (Figure 3).

For the clinical study, after approval by the Bioethics Committee of the University of Perugia, the project was presented to the schools (headmaster, teachers, parents, and pupils) for the recruitment of adolescents. The participants were given a swab for saliva collection with the relevant instructions. Tests were carried out for the study of the population, and questionnaires were administered to evaluate anxious and/or depressive symptoms. All the results obtained were correlated with each other. The in vitro study was performed to see if a specific *VDR* polymorphism genotype was important in the response to VitD treatment. For the in vitro study, three very different cell lines were chosen. In each cell line, the *Fok1* and *Apa1* polymorphic variants were evaluated, and a functional study was carried out in response to treatment with VitD. For this purpose, *VDR* mRNA expression and the expression of specific markers (NF200, vimentin, and HER2) were analyzed. The objective of the work was to lay the foundations for a possible correlation in the future between clinical data and in vitro data.

### 4.2. Study Population

A total of 184 adolescents aged 14 to 17 attending secondary schools in Umbria, Italy were recruited in this study from March 2022 to May 2023. The inclusion criteria included: Italian speakers, informed consent signed by both parents, non-clinical participants, and no family or friendship ties with the researcher (for ethical reasons and to avoid invalidating the evidence). Of the study population, <5% reported having consulted a psychologist for emotional problems in the past two years. Therefore, no participants were excluded due to psychiatric consultation or psychological counseling. The exclusion criteria were students older than that indicated, students repeating the school year, and those diagnosed with a mood disorder. Of the total number of participants, saliva was collected from 184 Italian adolescents with an average age of 15.15 years (*SD* = 1.02).

### 4.3. Ethics Approval and Informed Consent

The project was approved by the Bioethics Committee of the University of Perugia (n.69574), and all the procedures were performed accordingly. All the questionnaires were administered to the students by a psychologist from the University of Perugia. Participation in the study was on a voluntary basis, and parents signed informed consent for participation. The participants were anonymized, and no sensitive data were collected.

### 4.4. Assessment of Anxiety Symptoms

The DSM-5 Level 2 cross-sectional symptom rating scale (PROMIS Emotional Distress—Anxiety—Pediatric Item Bank; American Psychiatric Association, 2013), was used. This is a self-report questionnaire that assesses anxiety symptoms in adolescents aged 11 to 17, consisting of 13 items, the scores of which are rated on a 5-point Likert scale from 1 = never to 5 = almost always. Specifically, the questionnaire investigates how often, in the last 7 days, the different symptoms have been experienced by the subject. An example of an item is “I felt worried”. Four cut-offs are identified: (a) less than 55 = from absent to sporadic; (b) 55.0–59.9 = mild; (c) 60.0–69.9 = moderate; (d) 70.0 and over = severe. On this basis, for the present study, a specific cut-off was created: (a) less than 55 = absent; (b) over 55 = present.

### 4.5. Assessment of Depression Symptoms

For the assessment of depressive symptoms, the DSM-5 Level 2 Cross-Symptom Rating Scale was used (PROMIS Emotional Distress—Depression Pediatric Item Bank; American Psychiatric Association, 2013), a self-report questionnaire assessing depressive symptoms in adolescents aged 11 to 17 years, consisting of 14 items, the scores of which are rated on a 5-point Likert scale from 1 = never to 5 = almost always. Specifically, the questionnaire investigates how often in the last 7 days the subject experienced the different symptoms. An example of an item is “Being sad has made it difficult for me to do things with my friends”. Four cut-offs are identified: (a) less than 55 = from absent to sporadic; (b) 55.0–59.9 = mild; (c) 60.0–69.9 = moderate; (d) 70.0 and over = severe. On this basis, for the present study, a specific cut-off was created: (a) less than 55 = absent; (b) over 55 = present.

### 4.6. Collection of Saliva Samples

All the participants followed the same protocol for saliva sample collection. Specifically, specific disposable sterile pharmacy swabs (swabs) were used following detailed instructions such as: (1) carry out the sample on the same day as the sample is returned; (2) take the sample in the morning on an empty stomach without brushing your teeth; (3) before using the swab, make sure your hands are clean; (4) open the protective packaging and remove the swab; the part that touches the bottom of the container is the one that will be inserted into the mouth; (5) be careful not to drop the swab or place it anywhere; (6) place the swab in your mouth for 2 min, under your tongue and laterally along your cheeks, pressing vigorously from top to bottom; (7) remove the swab from your mouth; (8) reinsert the swab into the container; (9) replace the cap; (10) deliver the saliva sample the same morning. The samples were stored at −80 °C.

### 4.7. Genomic DNA Extraction

Genomic DNA was isolated from the saliva samples using a Monarch Genomic DNA Purification Kit (New England Biolabs, Ipswich, MA, USA). The gDNA was quantified using the NanoDrop system, and 200 ng of each sample was run in 0.8% agarose gel electrophoresis in 1× TBE buffer at 90 V for 45 min to verify the integrity of the gDNA.

### 4.8. Genotyping of Fok1 and Apo1 SNPs

Genotyping of *Fok1 rs2228570* and *Apa1 rs7975232* was performed using TaqMan SNP Genotyping Assays (Thermo Fisher Scientific, Waltham, MA, USA). Ten ng of the template gDNA was used for the assays. All the samples were analyzed with a 7500 Real-Time PCR (Applied Biosystem (Foster City, CA, USA)) apparatus using the recommended cycling conditions: a denaturation phase (95 °C for 15 min) followed by annealing and extension for 50 cycles (60 °C for 60 min), as previously described [37]. Each run was performed including positive controls (wild type, heterozygous, and homozygous variant genotypes). The specific dye-labeled probes were purchased by Applied Biosystems (Foster City, CA, USA): *Fok1* (rs2228570) forward primer (F): 5′-AGCTGGCCCTGGCACTGACTCTGGCTCT-3′ and reverse primer: 3′-ATGGAAACACCTTGCTTCTTCTCCCTC-5′; *Apa1* (rs7975232) forward primer: 5′-CAGAGCATGGACAGGGAGCAA-3′ and reverse primer: 3′-GCAACTCCTCATGGCTGAGGTCTC-5′.

### 4.9. Cell Culture and Treatments

The immortalized hippocampal neurons, HN9.10e, were the kind gift of Kieran Breen, Ninewells Hospital, Dundee, UK [38]. Human keratinocytes (HaCaT cell lines) were purchased from I.Z.S.L.E.R. from the Istituto Zooprofilattico Sperimentale della Lombardia e dell’Emilia Romagna ‘Bruno Ubertini’ (Brescia, Italy) [30]. The cell MDA–MCF7 line was produced by Elabscience Biotechnology (Houston, TX, USA) [34]. The cell lines were grown in Dulbecco’s modified Eagle’s medium (DMEM, MicrotechSrl, Pozzuoli, NA, Italy), supplemented with 10% fetal bovine serum (FBS), 2 mM of l-glutamine, 100 IU/mL of penicillin, and 100 μg/mL of streptomycin (Thermo Fisher Scientific, Waltham, MA, USA) Only for the HN9.10 cell lines, 2.5 μg/mL of amphotericin B (Invitrogen Srl, Milan, Italy) was added. The cell lines were maintained at 37 °C in a saturation humidity atmosphere containing 95% air and 5% CO_2_. After colonies were formed (80–90% confluence), the plates were washed with PBS 1× and harvested with 0.05% trypsin in 0.02% Na_4_EDTA for 10 min at 37 °C. Confluent cultures were passed every 2–3 days.

For all the experiments, six lots of HN9.10e, HaCat, and MCF7 cell lines at 1 × 10^5^/mL concentration were prepared. The cell lines were treated with 100 nM of 1α, and 25(OH)2VD3(VitD3) obtained from Sigma-Aldrich (now Merck, Darmstadt, Germany) was dissolved in absolute ethanol as the vehicle. In the control samples, only absolute ethanol was added in the same amount present in the experimental sample (50 µL/15 mL). After 24 h of culture, the cell lines were used for the *Fok1* and *Apa1* polymorphism, VDR gene expression, and immunocytochemistry analyses.

### 4.10. Quantitative Real-Time RT-PCR

The HN9.10e, HaCat, and MCF7 cell lines were used for total RNA extraction performed using the RNAqueous-4PCR kit (Ambion Inc., Austin, TX, USA), as previously reported [39]. RTqPCR was performed using Master Mix TaqMan Gene Expression and a 7500 RT-PCR instrument (Applied Biosystems, Foster City, CA, USA), targeting the *VDR* gene (Hs00172113_m) in a TaqManArray 96-well plate. Glyceraldehyde-3-phosphate dehydrogenase (*GAPDH*, Hs99999905_m1) and 18S rRNA (*S18*, Hs99999901_s1) were used as housekeeping genes. Relative mRNA expression levels were calculated as 2^−ΔΔCt^ by comparing the results of the VitD3-treated sample with those of untreated samples.

### 4.11. Immunocytochemistry

The cell block was obtained using the Hologic protocol for the Cellient Automated Cell Block System (Hologic, Marlborough, MA, USA) (Accessed on 28 April 2023) [https://www.hologic.com/sites/default/files/2020-07/MAN-06369–001_001_02.pdf]: in detail, the formalin-fixed cell cultures were first centrifuged at 1727 RPM for 10 min. After the removal of the supernatant, the cell precipitate was placed in a vial of Preservcyt solution (methanol-buffered solution) from the ThinPrep system (Hologic, Marlborough, MA, USA) and processed through Cellient instrumentation for 45 min, concentrating the cell lines and distributing them in a thin layer in a paraffin cell block. Subsequently, 4 µm sections were prepared and placed on positively charged slides for immunocytochemical staining using the fully automated BOND III immunohistochemical stainer (Leica Biosystems, Nußloch, Germany) [40]. Bond Dewax solution was used to remove paraffin from the sections before rehydration [41]. Immunostaining for NF200 and vimentin was performed by using specific antibodies; HER2 was detected using the HercepTest and Bond Polymer Refine Detection—Leica Biosystems (Newcastle, Ltd., Newcastle upon Tyne, UK). For NF200 and vimentin, 10 different areas of approximately 0.4 μ2 each were selected using QuPath 0.2.0-m9 software (Accessed on 28 April 2023) (https://github.com/qupath/qupath/releases?page=3) to automatically count the positive cell lines, the accuracy of which was then checked manually. Subsequently, the mean ± SD of the percentage values obtained was used to quantify the positivity of the staining. Regarding the evaluation of HER2 staining, a score from 0 to 3+ was applied, according to the current ASCO–CAP guidelines [33].

### 4.12. Statistical Analysis

For the statistical analyses, SPSS software (version 26) was used. Chi-square analysis was used to study: (1) the distribution of sex for the Fok1 and Apa1 genotypes; and (2) the distribution of genotypes in the subjects from Italian parents, foreign parents, a foreign mother, and a foreign father. Anova and *t*-tests for independent samples were used to assess: (1) differences in anxious and depressive symptoms in the different genotypes; and (2) differences in anxious and depressive symptoms in the genic models (allelic model, dominant model, and recessive model). For experiments in vitro, *t-*tests were used to compare the experimental samples (treated with VitD3) with the control samples (without treatments).

## 5. Conclusions

Our study demonstrates that there is no association between the occurrence of anxious and/or depressive symptoms in non-clinical adolescents and the *Fok1* and *Apa1* polymorphic variants of the *VDR* gene. However, 96.2% of the low percentage of adolescents presenting the GG–AA (Fok1/Apa1) genotype have both anxious and depressive symptoms. With the in vitro experiments, we demonstrated that cell lines with different *VDR* polymorphic variants respond differently to VitD3 treatment. In particular, cell lines with the GG–AA (*Fok1*/*Apa1*) genotype do not respond to VitD3 at all. The results are a starting point for further future investigations on the *VDR* polymorphic variant and the response to treatment with VitD3, both in the general population and in individuals with mental and/or neuropsychiatric disorders.

## Figures and Tables

**Table 1:** Total population and sex frequency distribution of *Fok1* and *Apa1* genotypes.

*Fok1* genotype								
	AA		GG		AG		Total	
	n	%	n	%	n	%	n	%
Total population	79	42.9	18	9.8	87	47.3	184	100
Males	30	38.0	5	27.8	33	37.9	68	37
Females	49	62.0	13	72.2	53	62.1	116	63
*Apa1* genotype								
	CC		AA		CA		Total	
	n	%	n	%	n	%	n	%
Total population	57	31	46	25	81	44	184	100
Males	24	42.1	17	37.0	27	33.3	68	37
Females	33	57.9	29	63.0	54	66.7	116	63

The analysis was performed in the total population (N = 184), males (N = 68) and females (N = 116). *Fok1* genotypes: AA, homozygous dominant genotype; GG, homozygous recessive genotype; AG, heterozygous genotype. *Apa1* genotypes: CC, homozygous dominant genotype; AA, homozygous recessive genotype; CA, heterozygous genotype. A chi-square analysis was performed. None of the analyses performed were significant.

**Table 2:** Distribution of genotypes for *Fok1* and *Apa1* in adolescents born to Italian parents, both foreign parents, a foreign mother, or a foreign father.

*Fok1* genotypes			
	AA	GG	AG
	%	%	%
Italian parents	40	9	51
Foreign parents	53	9	38
Foreign mother	34	7	59
Foreign father	64	9	27
Apa1 genotypes			
	CC	AA	CA
	%	%	%
Italian parents	31	24	45
Foreign parents	24	29	47
Foreign mother	28	28	44
Foreign father	18	32	50

*Fok1* genotypes: AA, homozygous dominant genotype; GG, homozygous recessive genotype; AG, heterozygous genotype. *Apa1* genotypes: CC, homozygous dominant genotype; AA, homozygous recessive genotype; CA, heterozygous genotype. Data are expressed as percentages of homozygous dominant, homozygous recessive, and heterozygous subjects. A chi-square analysis was performed. None of the analyses performed were significant.

**Table 3:** Depressive and anxious symptoms in relation to sex and age.

Age	Males				Females			
	Depression	CTR	Anxiety	CTR	Depression	CTR	Anxiety	CTR
total	25.0 *	75.0	30.9 *	69.1	67.2 *^§^	32.8	67.2 *^§^	32.8
14	11.8 *	88.2	35.3 *	64.7	70.6 *^§^	29.4	60.8 *^§^	39.2
15	7.1 *	92.9	7.1 *	92.9	68.2 *^§^	31.8	68.2 *^§^	31.8
16	38.5 *	61.5	30.8 *	69.2	57.9 *^§^	42.1	71.1 *^§^	28.9
17	36.4 *	63.6	54.5 *	45.5	100 *^§^	0	100 *^§^	0

Percentage of patients with depressive symptoms versus subjects without symptoms (CTR) and percentage of patients with anxious symptoms versus subjects without symptoms (CTR). Males and females were analyzed separately in the total population and in relation to age. The statistical analysis was performed by an ANOVA test and *t*-tests for independent samples. * *p* < 0.001 depression vs. CTR and * *p* < 0.001 anxiety vs. CTR in males and females. ^§^ *p* < 0.001 females with depression vs. males with depression and ^§^ *p* < 0.001 females with anxiety vs. males with anxiety.

**Table 4:** Presence of depressive and anxious symptoms in the same subject.

	Score Depression			Score Anxiety	
Anxiety	62.10 (9.57)	*p* < 0.001	Depression	62.42 (8.52)	*p* < 0.001
CTR	47.27 (8.53)		CTR	48.36 (7.57)	

Percentage of depressive symptoms in patients with anxiety and percentage of anxious symptoms in patients with depression. The statistical analysis was performed by an ANOVA test.

**Table 5:** Relationship between *Fok1* and *Apa1* polymorphic variants and the presence of anxious and/or depressive symptoms.

**a**							
**Model**	**Symptoms**	***Fok1***			***Apa1***		
		Genotype	F(2)	*p*	Genotype	F(2)	*p*
Allelic model	Depression	AA GG AG	1.000	0.370	CC AA AC	0.703	0.496
	Anxiety		0.029	0.971		2.300	0.103
**b**							
**Model**	**Symptoms**	***Fok1***			***Apa1***		
		Genotype	*t*	*p*	Genotype	*t*	*p*
Dominant model	Depression	AA + AG versus GG	−0.407	0.684	CC + CA versus AA	0.790	0.430
	Anxiety		0.162	0.872		1.897	0.059
Recessive model	Depression	GG + AG versus AA	−182	0.239	AA + CA versus CC	0.510	0.610
	Anxiety		−0.218	0.828		0.187	0.852

(a) Depression and anxiety symptoms analysis among an allelic model of *Fok1* and *Apa1* polymorphic variants in the total population under study (185 participants). F, ANOVA test; parenthesis shows degree of freedom. (b) Depression and anxiety symptoms in dominant or recessive models of *Fok1* and *Apa1* polymorphic variants in the total population under study (185 participants). *t*, Student’s *t*-test.

**Table 6:** *Fok1* and *Apa1* polymorphic variants in the HN9.10e, HaCat, and MCF7 cell lines.

Cell Line	*Fok1*	*Apa1*
HN9.10e	AA	CC
HaCat	AG	AC
MCF7	GG	AA

Genotypes of *Fok1* and *Apa1* in embryonic hippocampal cell lines (HN9.10e), keratinocytes (HaCat), and breast cancer cell lines (MCF7).

## Data Availability

No new data were created or analyzed in this study. Data sharing is not applicable to this article.
